# An anatomical study on lumbar arteries related to the extrapedicular approach applied during lumbar PVP (PKP)

**DOI:** 10.1371/journal.pone.0213164

**Published:** 2019-03-05

**Authors:** Liehua Liu, Shiming Cheng, Qian Wang, Qiang Liang, Yong Liang, Weidong Jin, Qiang Zhou, Zili Wang

**Affiliations:** 1 Department of Spinal Surgery, General Hospital of Ningxia Medical University, Yinchuan, Ningxia Hui Autonomous Region, China; 2 Department of Spine Surgery, The Third Affiliated Hospital of Chongqing Medical University (Gener Hospital), Chongqing, China; 3 Department of Orthopedics, Chongqing Dongnan Hospital, Chongqing, China; 4 Hillsborough Community College, Tampa, Florida, United States of America; 5 Department of Radiology, Southwest Hospital, The Army (Third Military) Medical University, Chongqing, China; Medical College of Wisconsin, UNITED STATES

## Abstract

To observe the regional anatomy of the lumbar artery (LA) associated with the extrapedicular approach applied during percutaneous vertebroplasty (PVP) and percutaneous kyphoplasty (PKP), we collected 78 samples of abdominal computed tomography angiography imaging data. We measured the nearest distance from the center of the vertebral body puncture point to the LA (distance VBPP-LA, D_VBPP-LA_). According to the D_VBPP-LA_, four zones, Zone I, Zone II, Zone III and Zone IV, were identified. LAs that passed through these zones were called Type I, Type II, Type III and Type IV LAs, respectively. A portion of the lumbar vertebrae had an intersegmental branch that originated from the upper segmental LA and extended longitudinally across the lateral wall of the pedicle; it was called Type V LA. Compared with the D_VBPP-LA_ in L1, L2, L3 and L4, the overall difference and between-group differences were significant (*P* < 0.05). In L1, L2, L3, L4 and L5, there were 8, 4, 4, 0 and 1 Type I LAs, respectively. There were no Type V LAs in L1 and L2, but there were 2, 16 and 26 Type V LAs in L3, L4 and L5, respectively. In L1-L5, the numbers of Type I LA plus Type V LA were 8, 4, 6, 16 and 27, and the presence ratios were 5.1%, 2.6%, 5.6%, 10.3% and 17.3%, respectively. In L4 and L5, the male presence ratios of Type I LA plus Type V LA were 7.1% and 10.7%, respectively, and the female presence ratios were 13.9% and 25.0%, respectively. Thus, extrapedicular PVP (PKP) in lumbar vertebrae had a risk of LA injury and was not suggested for use in L4 and L5, especially in female patients.

## Introduction

In 2005, Han *et al*. [1] reported extrapedicular percutaneous vertebroplasty (PVP) in the treatment of thoracic vertebral compression fracture. In 2007, Ryu *et al*. [2] reported the surgical technique and clinical effects of extrapedicular percutaneous kyphoplasty (PKP) with a single balloon in 13 lumbar vertebrae. In 2011, Cho *et al*. [3] reported extrapedicular PVP and PKP in 74 lumbar vertebrae, confirming the efficacy and feasibility of the extrapedicular approach for lumbar PVP (PKP). The main advantage of the extrapedicular approach is that the puncture needle can easily reach the midline of the vertebral body to facilitate bone cement diffusion in the central part of the vertebral body. Moreover, bilateral puncture is not needed, reducing trauma and saving operative time. In addition, an extrapedicular approach puncture can maintain the integrity of the pedicle cortex, preserving the axial or lateral biomechanical stability of the spine. [4]

In the puncture technique of the extrapedicular approach, the puncture needle must cling to the lateral side of the upper articular process and the upper edge of the basal transverse process and penetrate the vertebral body at the junction between the lateral side of the pedicle and the posterolateral side of the vertebral body. [5] However, the lumbar artery (LA) is distributed on the posterolateral side of the vertebral body, and the intersegmental branch passes through the lateral side of the pedicle that comes from the upper segmental LA; therefore, the extrapedicular approach carries a risk of LA injury. [6–9] Heo *et al*. [7] reported a case of a 73-year-old female patient with severe radiological pain and tingling in the left leg after L2 PVP. The systolic pressure was reduced from 130 mmHg to 95 mmHg at 6 hours postoperatively, computed tomography (CT) scans showed a large retroperitoneal hematoma, and angiography confirmed bleeding in the left 2^nd^ LA. Subsequently, a microcoil and gelatin sponge embolism was applied, and 500 ml of the liquefied hematoma was suctioned under ultrasound guidance on the 40^th^ postoperative day. In 2006, Biafora *et al*. [8] reported an extrapedicular PKP in an L5 compressed fracture. On the 10^th^ postoperative day, an incision hemorrhage occurred, and hemoglobin decreased from 9.3 g/dL to 8.2 g/dL in 4 hours. Selective angiography confirmed intersegmental branch bleeding; this branch originated from the right 4^th^ LA, and an embolism was used to successfully control the bleeding.

The extrapedicular puncture path is adjacent to the LA; thus, understanding the trend and distribution of the LA in the posterolateral vertebral body and the lateral side of the pedicle is very important. At present, anatomical studies related to LAs mainly focus on extreme lateral lumbar interbody fusion, [10] lumbar discectomy, [11] and flap transplantation with LA perforators. [12] However, within the scope of the literature, there is no systematic study on the anatomy of the LA associated with the extrapedicular approach applied during lumbar PVP (PKP).

Therefore, the authors used computed tomography angiography (CTA) to observe the regional anatomy of the LA associated with the extrapedicular approach applied during lumbar PVP (PKP) and analyzed the clinical significance to aid in evaluation of the risk of LA injury during extrapedicular puncture.

## Materials and methods

### General information

From September 2017 to December 2017, imaging data were collected from 78 adults who underwent total abdominal CTA because of urinary or intestinal diseases. No samples had lumbar scoliosis, lumbar spondylolisthesis, lumbar bone destruction, lumbar fracture, LA disease, or urinary and lumbar surgery history. All methods in this study were performed in accordance with the Helsinki Declaration’s relevant guidelines and regulations. All experimental protocols were approved by the Scientific Research Ethics Committee of Ningxia Medical University General Hospital. All data were fully anonymized before being obtained for use in this retrospective study. Written informed consent was obtained from all enrolled participants.

### Methods

**Fig 1:**
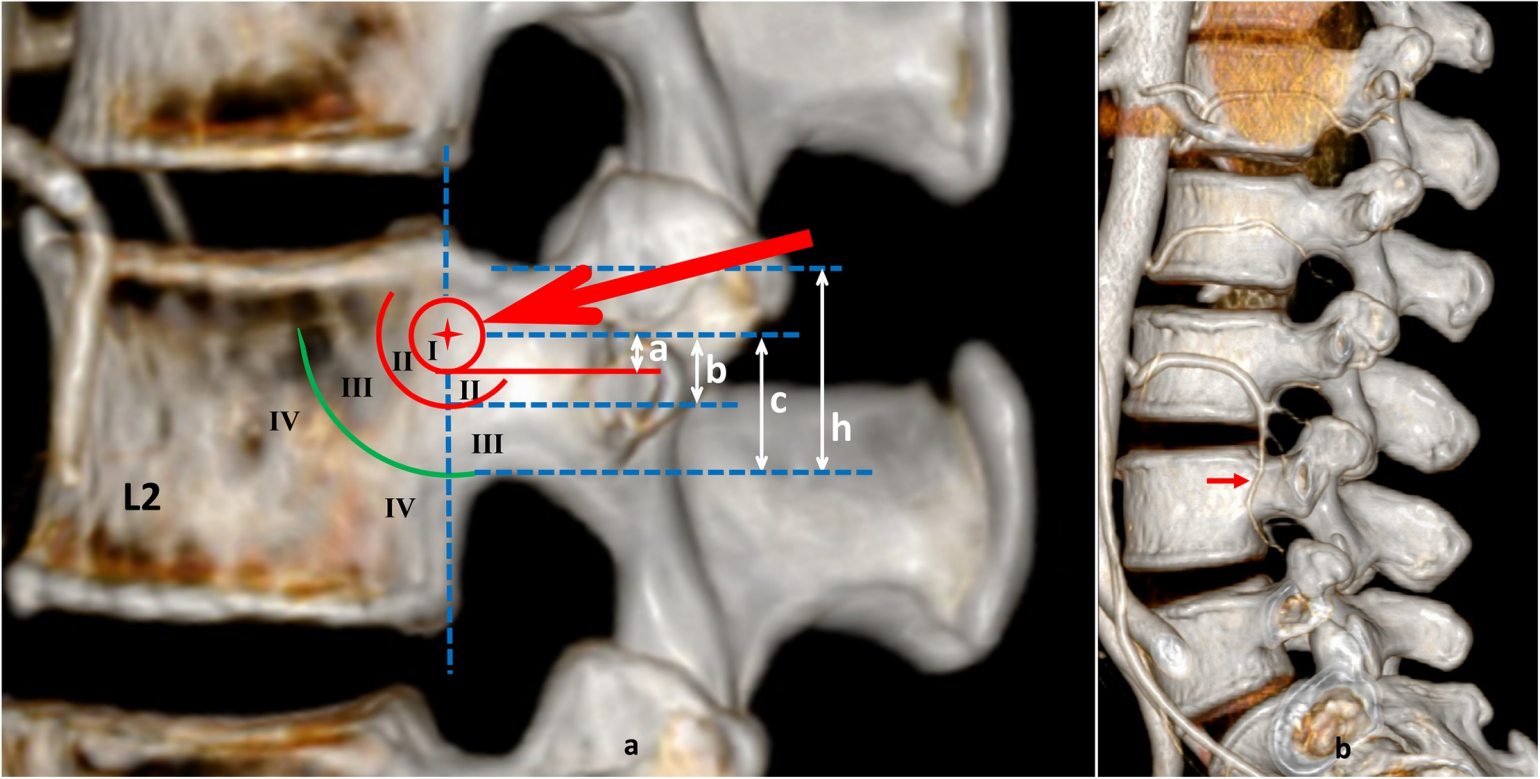
Zone I-IV in the posterolateral side of the vertebral body and intersegmental branch. a, circle: the vertebral body puncture point, diameter 5 mm. Arrow: puncture path. h: the sagittal pedicle isthmus width. Longitudinal dashed line: L2 vertebral body posterior border. Horizontally dashed lines: dividing h into 3 equal parts. a = 0.25 mm, b = 5 mm, c = 10 mm, h = 15 mm. I, II, III and IV represent Zone I, Zone II, Zone III and Zone IV, respectively. b, the intersegmental branch in L4 that originated from the left 3^rd^ LA and longitudinally passed through the lateral wall of the L4 left pedicle.

A Somatom Definition dual-source spiral CT (SIEMENS Corporation, Munich, Germany) scanner was used to image the entire abdomen, including the total lumbar vertebrae and LAs. The slice thickness was 5 mm, the pitch was 1.15 mm, the reconstructive slice thickness was 1 mm, and the overlapping rate was 30%. Contrast agent (Omnipaque) was injected into the median cubital vein, with a dose of 100 ml (100 ml: 35 g I). The injection rate was 4 ml/s. The scan time was 25 s to 30 s in the arterial phase and 60 s to 70 s in the venous phase. All images were subjected to maximum intensity projection, volume rendering and multiplanar reformation to clearly show the large vascularization and LAs. The postprocessing workstation of a SIEMENS dual-source spiral CT was used to observe the anatomy of LAs in each lumbar vertebra (Fig 1). First, we observed the LA origin, number, trend, absence and intersegmental branches on the reconstructed image.

Second, we measured the nearest distance from the center of the vertebral body puncture point to the LA (Distance VBPP-LA, D_VBPP-LA_) on the left and right lateral of the reconstructed image. The vertebral body puncture point was located at the junction between the middle-upper 1/3 of the lateral wall of the pedicle and the posterolateral side of the vertebral body (Fig 1A). In Fig 1, the circle is the vertebral body puncture point with a diameter of 5 mm and is called Zone I. According to the D_VBPP-LA_, the areas in front of Zone I and under Zone I were divided into Zone II (5 mm > D_VBPP-LA_ ≥ 2.5 mm), Zone III (10 mm > D_VBPP-LA_ ≥ 5 mm) and Zone IV (D_VBPP-LA_ ≥ 10 mm). Data measurements were performed independently by two spine surgeons, and the averaged values were used. Measurements were taken again if the measurement distance was different by ≥ 3 mm.

Finally, the LAs of each lumbar vertebra were classified into four types according to the different zones as follows: Type I LA, which passed through Zone I; Type II LA, which passed through Zone II; Type III LA, which passed through Zone III; and Type IV LA, which passed through Zone IV. A portion of the lumbar vertebrae did not have a LA that extended from the front to the back. However, there might be an intersegmental branch that extended longitudinally across the lateral wall of the basal pedicle that originated from the upper segmental LA and was located at the puncture path (Fig 1B). This intersegmental branch was called Type V LA.

### Statistical analysis

SPSS 19 software was employed for statistical analyses. Anatomical parameters are presented as the $\bar{x}$ ± S. Unpaired *t* test was used to analyze differences between males and females if the measurement data were normally distributed; if not, a Mann-Whitney test was used. One-way analysis of variance was used to analyze differences within the same parameter among the five lumbar levels. *P* < 0.05 was considered statistically significant.

## Results

**Fig 2:**
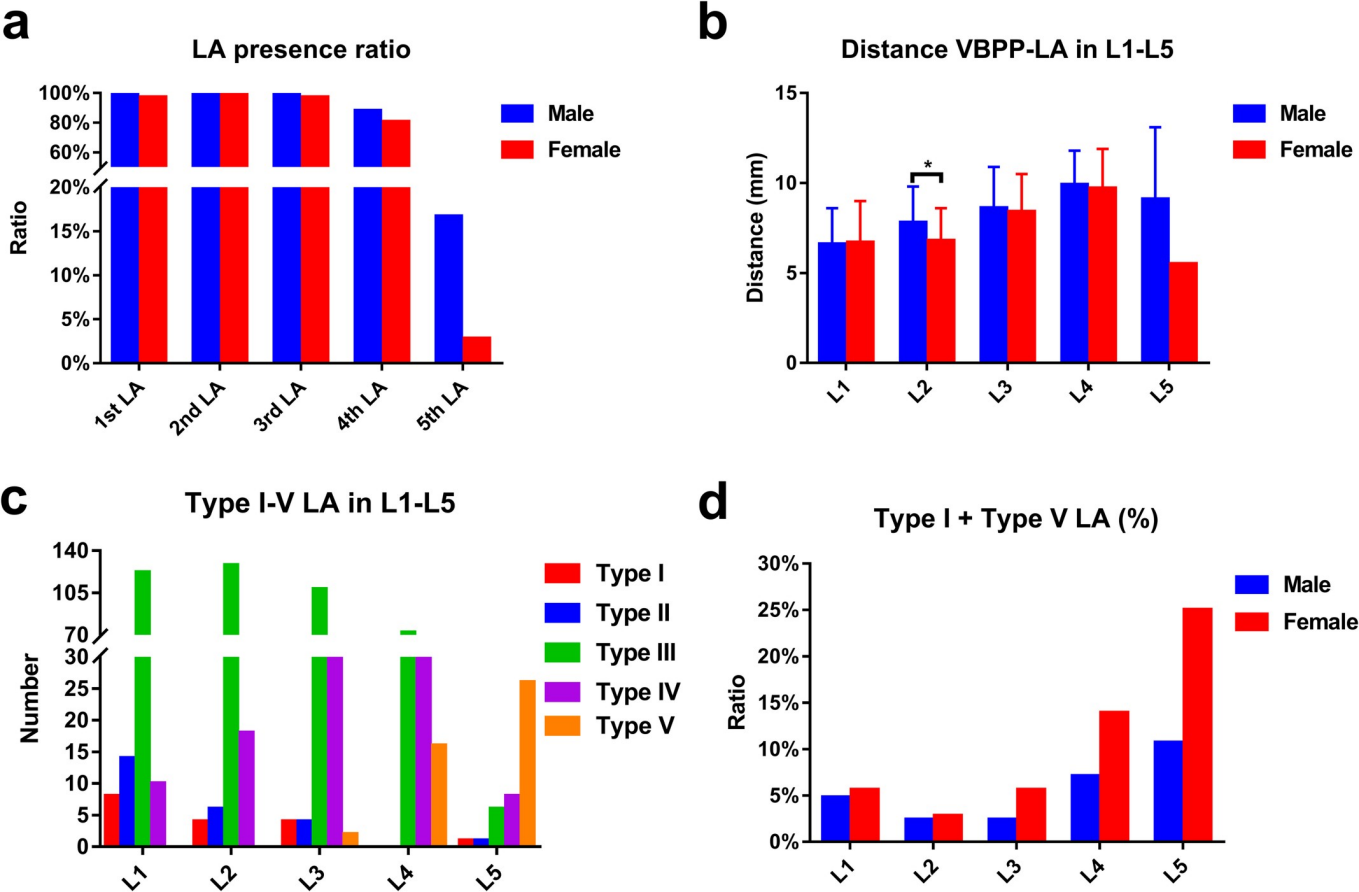
LA presence ratio, DVBPP-LA, and Type I-V LA in L1-L5. a, the presence ratio of LAs in L1-L5. b, D_VBPP-LA_ comparison between males and females in L1-5, * represents *P* < 0.05. c, the number of Type I-V LAs. d, the male and female ratios of Type I LA plus Type V LA in L1-L5.

**Fig 3:**
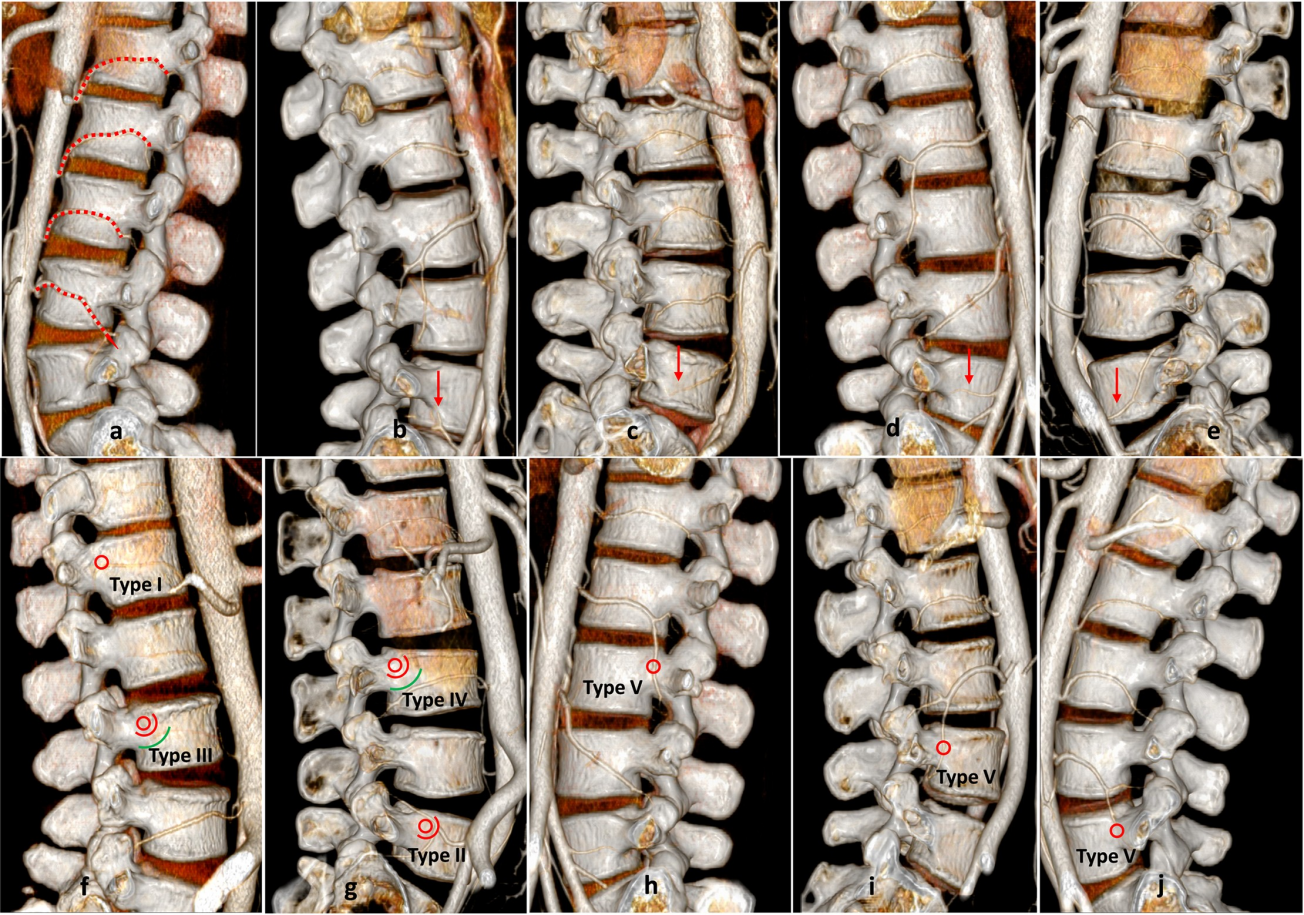
The origin of the 5^th^ LA and Type I-V LA. a, LA on the lateral side of the vertebral body in L1-L4. b, the 5^th^ LA originated from the iliolumbar artery. c, the 5^th^ LA originated from the abdominal aorta. d, the 5^th^ LA originated from the internal iliac vein. e, the 5^th^ LA originated from the common iliac artery. f, Type I LA in L1, Type III LA in L3. g, Type II LA in L5, Type IV LA in L3. h, Type V LA in L3. i, Type V LA in L4. j, Type V LA in L5.

This study included 42 males and 36 females, aged 44.0 ± 10.5 years old (20–70 years). The average male age was 45.7 ± 11.7 years old (22–70 years), and the average female age was 42.0 ± 8.7 years old (20–61 years). Age was not significantly different between males and females (*P* = 0.112). The LA number and absence, intersegmental branches and D_VBPP-LA_ in each lumbar vertebra are shown in S1 Table and Table 1 (Fig 2A and 2B). The 1^st^ and 2^nd^ LA origin were mostly parallel to the upper edge of the inferior vertebral body, and the 3^rd^ LA origin was mostly parallel to the L3/4 intervertebral space. LAs in L1-L3 inclined on the outward and upward sides of the vertebral body. The 4^th^ LA origin was in front of the middle part of the L4 vertebral body, which passed backward and downward through both sides of the L4 vertebral body (Fig 3A). In L1 and L2, LAs entered intervertebral foramens in the anterior and superior positions of the intervertebral foramen, while in L3 and L4, LAs entered intervertebral foramens in front of the intervertebral foramen. The presence ratios of LAs in L1-L3 were greater than 98%, and the ratio in L4 was 84.6%, indicating that 24 LAs were missed. Only 16 (10.3%) LAs were observed in L5, and 8 of these LAs originated from the iliolumbar artery, 4 from the abdominal aorta, 3 from the internal iliac artery and 1 from the common iliac artery (Fig 3B, 3C, 3D and 3E). In the absence of normal LAs, an intersegmental branch appeared in some lumbar vertebrae. The numbers of intersegmental branches in L3, L4 and L5 were 2, 16 and 26, respectively. The D_VBPP-LA_ in L2 was significantly different between males and females (*P* = 0.001), but the D_VBPP-LA_ in L1, L3 and L4 was not significantly different between males and females (*P* > 0.05) (Fig 2B). Compared with the D_VBPP-LA_ in L1, L2, L3 and L4, the overall difference and between-group differences were significant (*P* < 0.05). In L5, a smaller number of LAs were observed, and only 2 branches were observed in females. Thus, the D_VBPP-LA_ in L5 was not included in comparative statistical analysis.

**Table 1:** Basic data on LAs and D_VBPP-LA_ in L1-L5.

	Branches		Absence		Intersegmental branch		D_VBPP-LA_ (mm)
	n	%	n	%	n	%	
L1							
Male	84	100.0%	0		0		6.6±2.0
Female	70	97.2%	2	2.8%	0		6.7±2.3^Δ^
Male +female	154	98.7%	2	1.3%	0		6.6±2.1
L2							
Male	84	100.0%	0		0		7.8±2.0
Female	72	100.0%	0		0		6.8±1.8*
Male +female	156		0		0		7.3±2.0
L3							
Male	84	100.0%	0		0		8.6±2.3
Female	70	97.2%	2	2.8%	2	2.8%	8.4±2.1^Δ^
Male +female	154	98.7%	2	1.3%	2	1.3%	8.5±2.2
L4							
Male	74	88.1%	10	11.9	6	7.1%	9.9±1.9
Female	58	80.6%	14	19.4%	10	13.9%	9.7±2.2^Δ^
Male +female	132	84.6%	24	15.4%	16	10.3%	9.8±2.0
L5							
Male	14	16.7%	70	83.3%	8	9.5%	9.1±4.0
Female	2	2.8%	70	97.2%	18	25.0%	5.5±0.0
Male +female	16	10.3%	140	89.7%	26	16.7%	8.7±3.9

*, represents a comparison between males and females, *P* < 0.05; Δ, represents a comparison between males and females, *P* > 0.05.

The numbers of Type I-V LAs in L1-L5 are shown in Table 2 (Fig 2C, Fig 3F, 3G, 3H, 3I and 3J). In L1, Type III LA occurred most often, followed by Type II LA. In L2, L3 and L4, Type III occurred most often, followed by Type IV. In L1, L2, L3, L4 and L5, there were 8, 4, 4, 0 and 1 Type I LAs, respectively. There were no Type V LAs in L1 and L2, and there were 2, 16 and 26 Type V LAs in L3, L4 and L5, respectively. In L1-L5, the numbers of Type I LA plus Type V LA were 8, 4, 6, 16 and 27, respectively, and the presence ratios were 5.1%, 2.6%, 5.6%, 10.3% and 17.3%, respectively. In L4 and L5, the male presence ratios of Type I LA plus Type V LA were 7.1% and 10.7%, respectively, and the female presence ratios were 13.9% and 25.0%, respectively (Fig 2D).

**Table 2:** The number of Type I-V LAs and Type I + Type V LAs in L1-L5.

	Type I LA		Type II LA		Type III LA		Type IV LA		Type V LA		Type I + Type V LA	
	n	%	n	%	n	%	n	%	n	%	n	%
L1												
Male	4	4.8%	6	7.1%	69	82.1%	5	6.0%	0		4	4.8%
Female	4	5.6%	8	11.1%	53	73.6%	5	6.9%	0		4	5.6%
Male + female	8	5.1%	14	9.0%	122	78.2%	10	6.4%	0		8	5.1%
L2												
Male	2	2.4%	0		68	81.0%	14	16.7%	0		2	2.4%
Female	2	2.8%	6	8.3%	60	83.3%	4	5.6%	0		2	2.8%
Male + female	4	2.6%	6	3.8%	128	82.1%	18	11.5%	0		4	2.6%
L3												
Male	2	2.4%	2	2.4%	56	66.7%	24	28.6%	0		2	2.4%
Female	2	2.8%	2	2.8%	52	72.2%	14	19.4%	2	2.8%	4	5.6%
Male + female	4	2.6%	4	2.6%	108	69.2%	38	24.4%	2	1.3%	6	3.8%
L4												
Male	0		0		36	42.9%	38	45.2%	6	7.1%	6	7.1%
Female	0		0		36	50.0%	22	30.6%	10	13.9%	10	13.9%
Male + female	0		0		72	46.2%	60	38.5%	16	10.3%	16	10.3%
L5												
Male	1	1.3%	1	1.3%	4	4.8%	8	9.5%	8	9.5%	9	10.7%
Female	0		0		2	2.8%	0		18	25.0%	18	25.0%
Male + female	1	0.6%	1	0.6%	6	3.8%	8	5.1%	26	16.7%	27	17.3%

## Discussion

LA injury is more common in abdominal trauma and knife stab wounds, [13–14] but there has been no lack of reports on iatrogenic LA injury, which is more common in spine surgery [6–9,15–18] and urinary surgery. [19–20] In a previous meta-analysis, the surgical technical complication incidence associated with PVP (PKP) ranged from 1.8% to 3.8%. [21–22] However, vascular complications associated with PVP (PKP), such as aortic adventitial injury, [23] infection-induced aortic aneurysm [24] and LA injury, [6–9] have not been systematically documented. Giordano *et al*. [6] reported a ruptured LA pseudoaneurysm after PVP and cryoablation for a L2 metastatic tumor, resulting in a sudden and large number of retroperitoneal hemorrhages and hypovolemic shock. The authors noted that it was necessary to preoperatively confirm the anatomy of LAs through enhanced CT and puncture path design. Ajit *et al*. [9] reported two cases of 3^rd^ LA pseudoaneurysms after PVP and suggested that it was very important to master the bony anatomical landmarks and the vascular region of the vertebral body through clear fluoroscopy, especially being alert to LA injury during the extrapedicular approach puncture.

LAs are mostly paired small blood vessels that originate from the abdominal aorta and pass posteriorly to both sides of lumbar vertebrae. These arteries may have an unusual anatomical location. [25] Heo *et al*. [7] thought that the extrapedicular approach puncture may damage the segmental artery; this artery is difficult to monitor and detect during PVP (PKP) surgery. However, the limited number of reports related to LA injury does not mean that LAs have a low injury ratio that is not noticed clinically or discovered in time. In clinical practice, a small number of patients complained that their pain was not significantly relieved after PVP (PKP), but the pain location, characteristics and intensity were inconsistent with preoperative pain following a vertebral fracture. After bed rest and analgesics, the pain gradually decreased. In this situation, LA injury was not completely excluded because X-rays were performed after PVP (PKP) and generally did not include CT, magnetic resonance imaging (MRI) and CTA, resulting in a low discovery ratio of LA injuries. Unless the patient had a large amount of bleeding that caused a change in blood volume or stimulated the nerve root, LA injuries were not easily found. [7–8]

The safe puncture area in the vertebral body during extrapedicular PVP (PKP) in lumbar vertebrae should be slightly higher than the sagittal midline of the pedicle to avoid segmental artery injury. [7] The sagittal pedicle isthmus width was approximately 15 mm; [26] thus, in this study, the puncture point of the vertebral body during extrapedicular puncture was located at the middle-upper 1/3 of the lateral wall of the pedicle that was connected to the vertebral body. The common working channel diameter was 4.2 mm for PVP (PKP) and 5.2 mm for Sky PVP. Therefore, the diameter of the puncture point on the vertebral body was approximately 5 mm. In theory, LAs located at the puncture point on the vertebral body would be damaged. A greater V_BPP-LA_ distance resulted in a lower likelihood of LA damage. According to the positional relationship between LAs and the vertebral body puncture point, the area where LAs passed through was divided into 4 zones. Next, these LAs were classified into 4 types. Type I LA, passing through the vertebral body puncture point, was easily damaged. The injury risk of Type II LA was high, the injury risk of Type III LA was low, and the injury risk of Type IV LA was relatively low.

In some lumbar vertebrae without LAs, there was an intersegmental branch across the lateral wall of the basal pedicle that originated from the upper LA. The authors called this intersegmental branch Type V LA; it mostly appeared in L4 and L5. Type V LA was easily damaged as well as Type I LA because it longitudinally passed through the lateral side of the basal pedicle, which was located within the puncture path. Cho *et al*. [3] thought that there were no major blood vessels outside the pedicle by observation in only 2 cadavers; thus, the authors arbitrarily concluded that extrapedicular lumbar PVP (PKP) was feasible. As the sample size was too small to be representative, their results were inconsistent with those of this study. In the present study, in L1, L2, L3, L4 and L5, the presence ratio of Type I LA plus Type V LA was 5.1%, 2.6%, 5.6%, 10.3% and 17.3%, respectively. The presence ratio of Type I LA plus Type V LA indicated the occurrence ratio of LA injury during the extrapedicular approach for PVP (PKP). In L5, the occurrence ratio of LA injury during the extrapedicular approach for PVP (PKP) was the highest, reaching 17.3% (male 10.7%, female 25.0%), followed by L4 in which the occurrence ratio reached 10.3% (male 7.1%, female 13.9%).

Therefore, the authors did not suggest the use of extrapedicular PVP (PKP) for L4 and L5, especially for female patients. First, the LA was located in the puncture path, resulting in a high possibility of injury. Second, the intersegmental branch mostly originated from the 3^rd^ or 4^th^ LA, with a larger diameter. [25] Once the LA was damaged, bleeding was not easily stopped. Finally, the transverse pedicle isthmus width in L4 and L5 was 12.9 mm and 18.0 mm, respectively; [26] thus, the puncture needle had a sufficient abduction angle to achieve a satisfactory puncture during the transpedicular approach. [27–28]

Notably, in the present study, the sample comprised LAs in a normal physiological state. In a lumbar compression fracture, the distance from the LA to the vertebral body puncture point would be closer, increasing the risk of LA injury. Specifically, in a severe vertebral compression fracture, the height of the vertebral body was compressed to no more than 1/3 of the original height. [29] Because the transpedicular approach could lead to perforation of the injured vertebral endplates, an extrapedicular approach was selected. [30] To penetrate the puncture needle into the appropriate place in the vertebral body, the position of the vertebral body puncture point should be selected in the midline of the pedicle or even lower. [31] However, in severe vertebral compression fractures, Type II LA, originally located in Zone 2, and Type III LA, originally located in Zone 3, might be located at the puncture injury zone or adjacent to the puncture injury zone during the extrapedicular approach. Thus, the high proportion of Type II and Type III LAs greatly increased the risk of LA injury.

In addition, the extrapedicular approach has often been used during vertebral biopsy in clinical practice; some reports have associated this approach with LA injury. [17–18] Hence, the results of this study have a certain guiding significance for the extrapedicular approach in vertebral biopsy. However, this study also has certain limitations, such as a small sample size and some inevitable measurement errors.

### Conclusion

Extrapedicular PVP (PKP) in lumbar vertebrae had a certain risk of LA injury and is not suggested for use in L4 and L5, especially in female patients.

## Supporting information

S1 Table Study data. **D**_**VBPP-LA**_ **data in L1-L5 for each sample.** Absence indicates that the LA was absent. Absence (ISB) indicates that the LA was absent, but there was an intersegmental branch originating from the upper LA. * represents the 5^th^ LA originated from the iliolumbar artery. # represents the 5^th^ LA originated from the internal iliac artery. & represents the 5^th^ LA originated from the common iliac artery. Red table cell: Type I LA; yellow table cell: Type II LA; blue table cell: Type III LA; green table cell: Type IV LA; ISB: Type V LA.
(XLSX)
